# Supplementary material for: Genomic factors contributing to the resilience of Salmonella enterica on ready-to-eat muskmelon
Source: Food Microbiol. Author manuscript; Available in PMC 2026 Mar 1. (PMC12767474; doi:10.1016/j.fm.2025.104947)
Supplement: MMC 1 [file NIHMS2127750-supplement-MMC_1.docx]

**Supplementary Figure S1. Growth of the *Salmonella enterica* barcoded transposon mutant libraries (lib) and the corresponding wild types (wt) on ready-to-eat muskmelon at 22°C (A) and 8°C (B).** Growth curves at 22°C (A) with sampling time points from the inoculum, and at t_1_ (1 h incubation), t_7_ (7 h) and t_24_ (24 h), and 8°C (B) with sampling time points from the inoculum and from d_1_ (1 h of incubation) to d_5_ (96 h) every 24 h. Screenings were performed in PBS on RTE muskmelon (m) and in only PBS (negative controls; nc). STM: *S.* Typhimurium strain ATCC 14028; SEN: *S.*Enteritidis PT4 strain P125109; *S.* Newport C4.2.

*Growth of SNP_lib and SNP_wt significantly differ from each other at time point d_1_.
