# Supplementary material for: Genomic factors contributing to the resilience of Salmonella enterica on ready-to-eat muskmelon
Source: Food Microbiol. Author manuscript; Available in PMC 2026 Mar 1. (PMC12767474; doi:10.1016/j.fm.2025.104947)
Supplement: MMC 3 [file NIHMS2127750-supplement-MMC_3.docx]

Supplementary Figure S3. Quantitative assessment of the background microbiota on the ready-to-eat muskmelon samples. Mesophilic aerobic bacteria (MAB) and enterobacteria were enumerated at 22°C **(Panel A)** at the following time points: 1 h of incubation (t_1_), 7 h (t_7_), and 24 h (t_24_). Enumeration at 8°C (Panel B) was performed from day 1 (d_1,_ after 1 h of incubation) through day 5 (d_5_, 96 h) every 24 h. Data represents the means of five biological replicates, each with two technical replicates. Error bars indicate the standard deviation.
