# Supplementary material for: Genomic factors contributing to the resilience of Salmonella enterica on ready-to-eat muskmelon
Source: Food Microbiol. Author manuscript; Available in PMC 2026 Mar 1. (PMC12767474; doi:10.1016/j.fm.2025.104947)
Supplement: MMC 4 [file NIHMS2127750-supplement-MMC_4.docx]

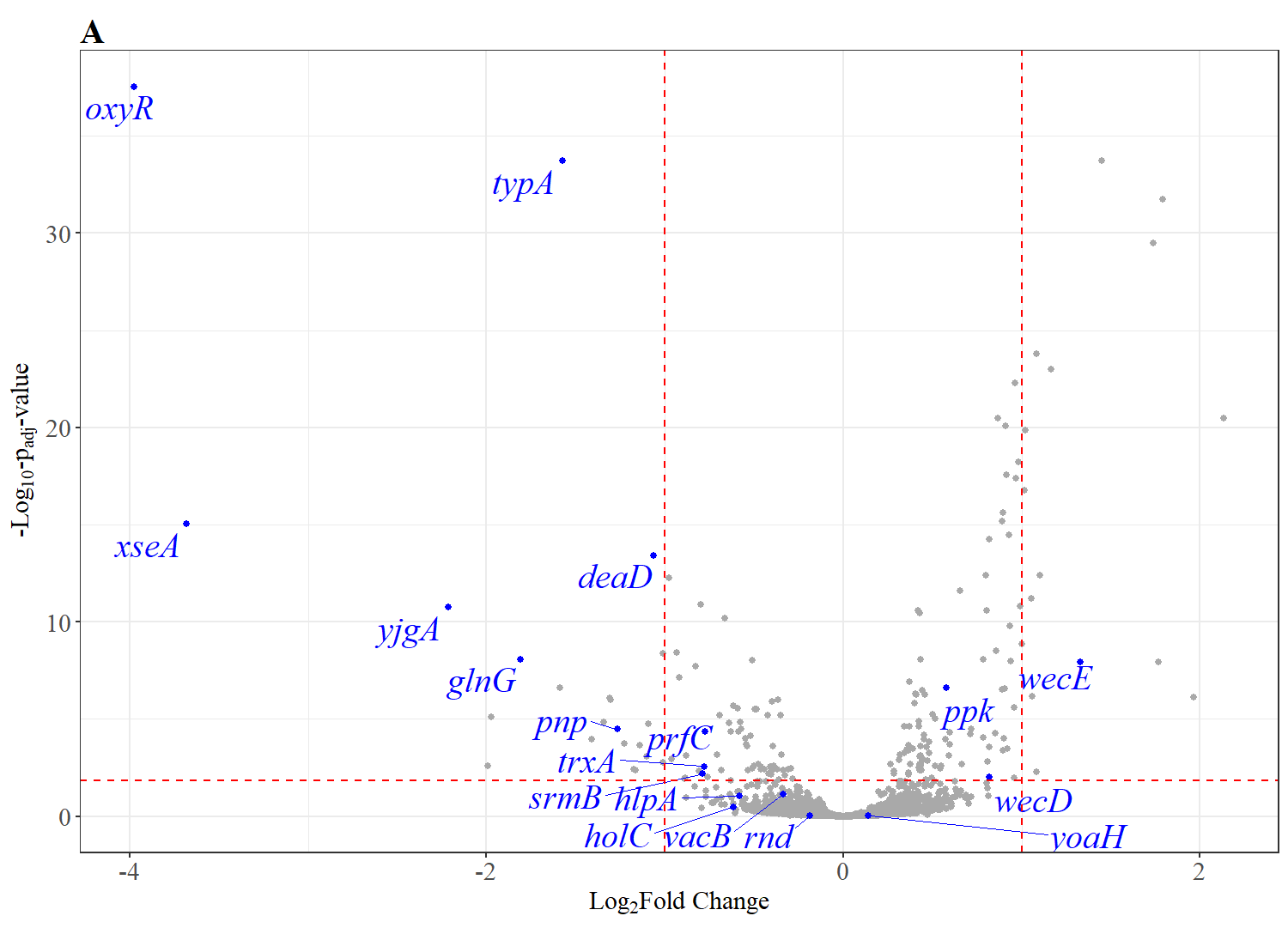


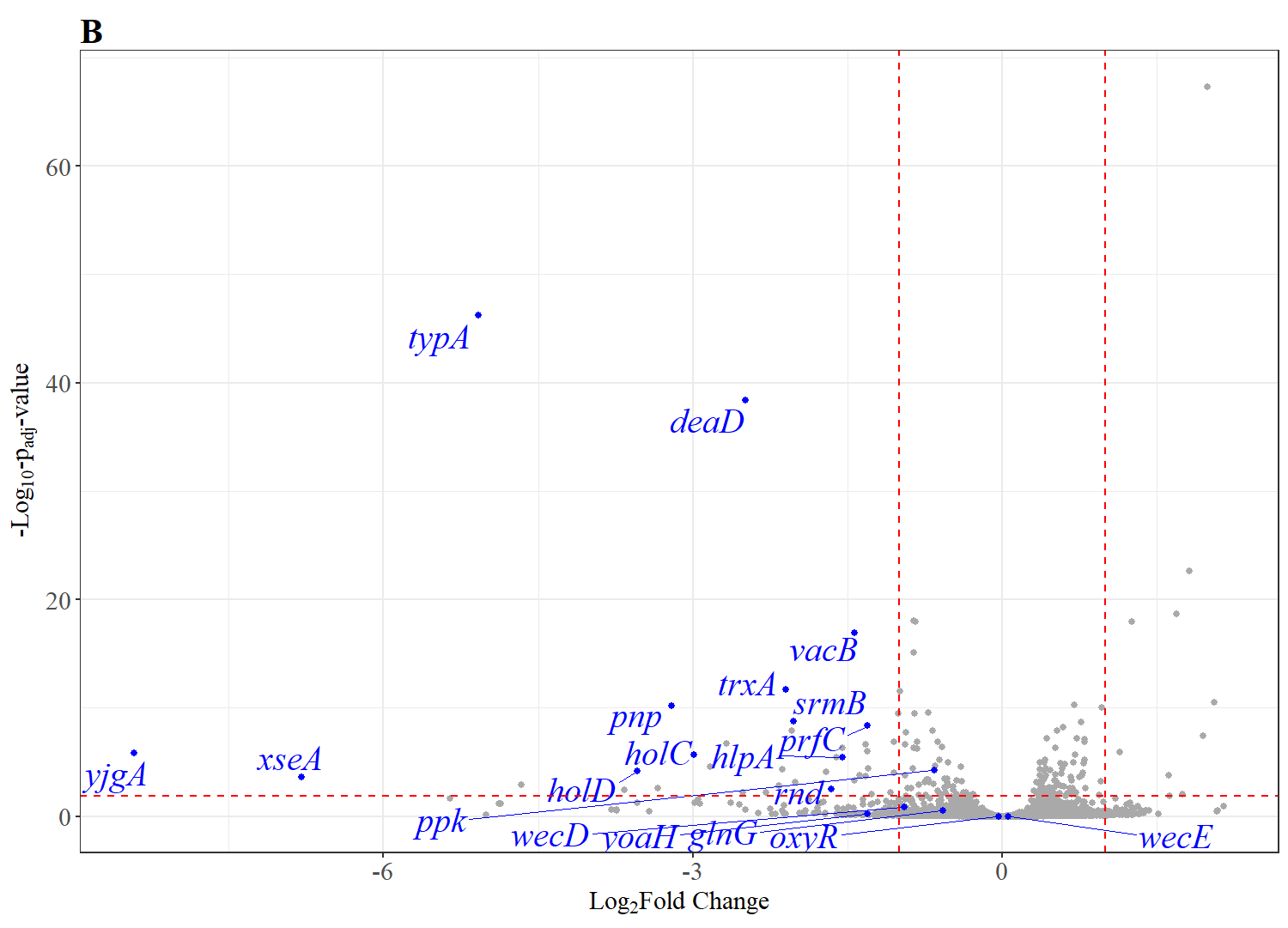


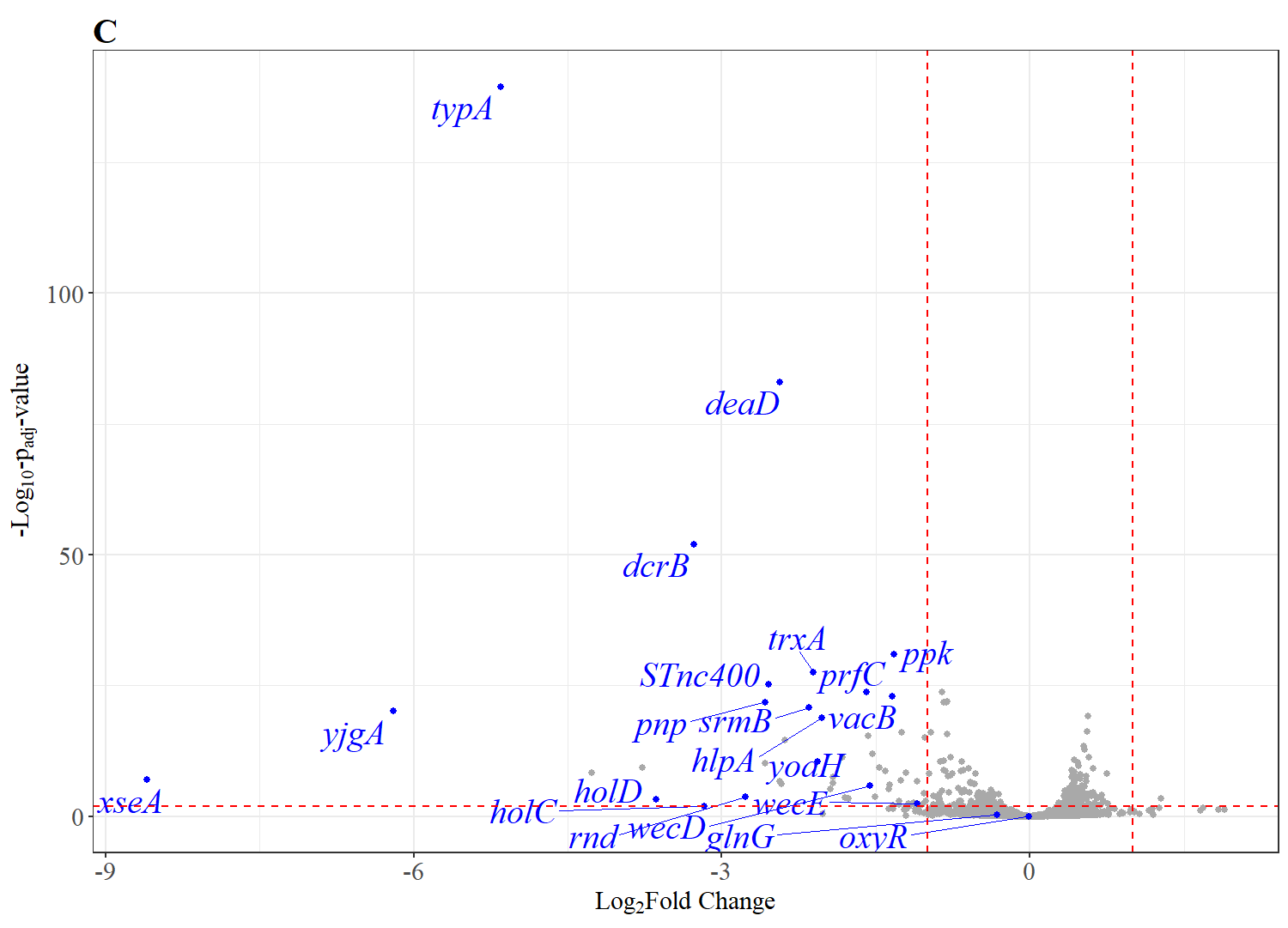


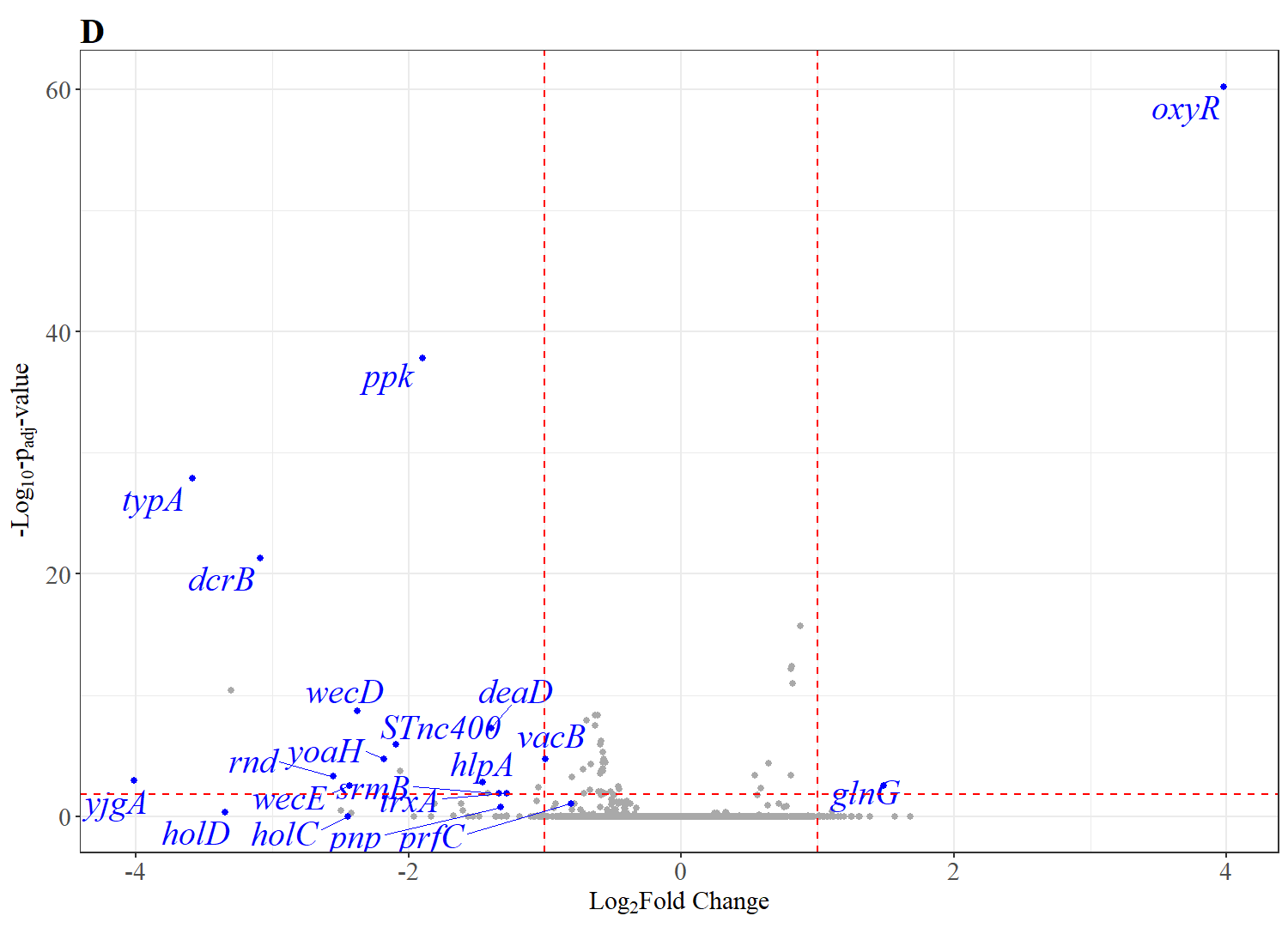


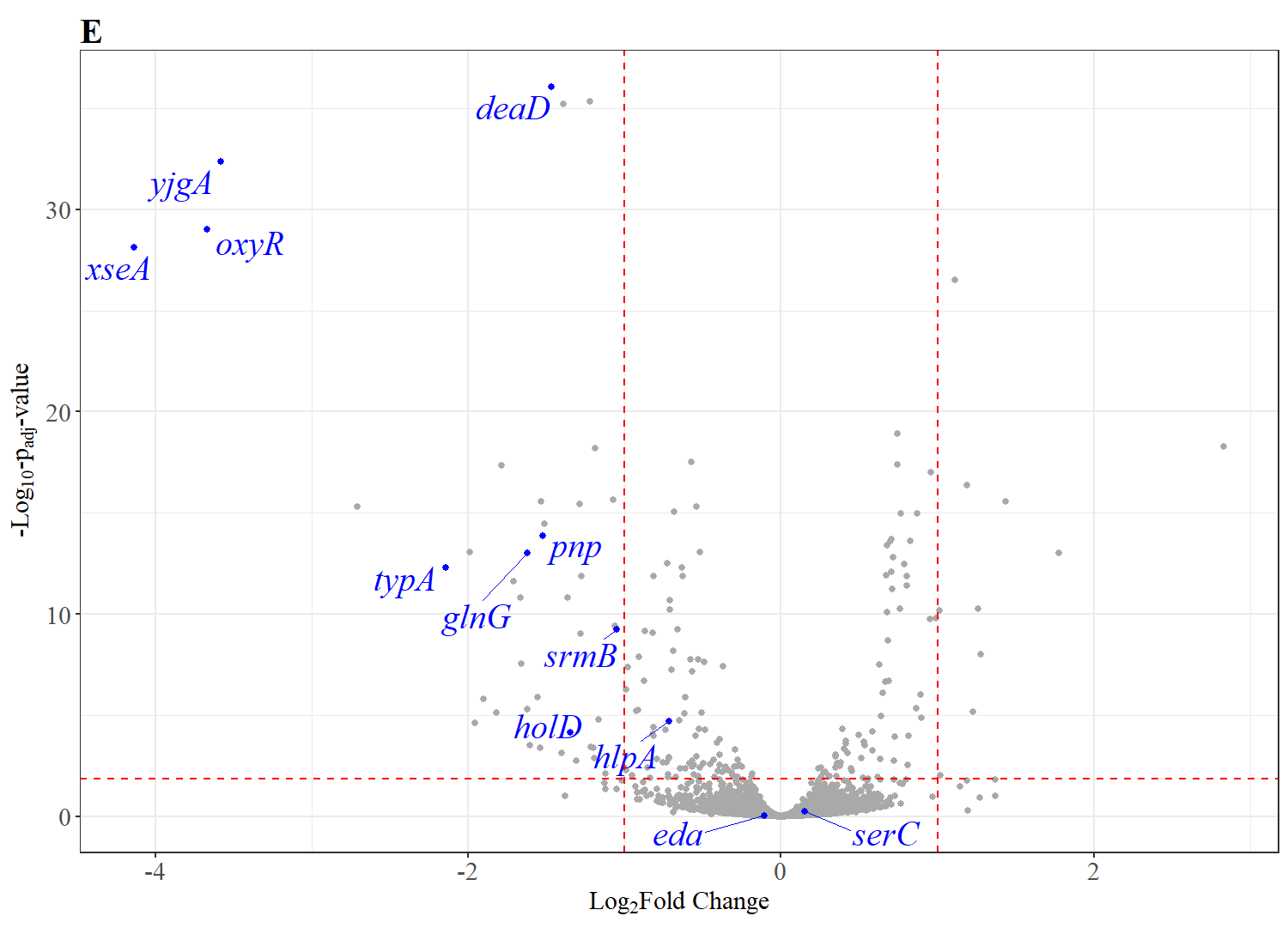


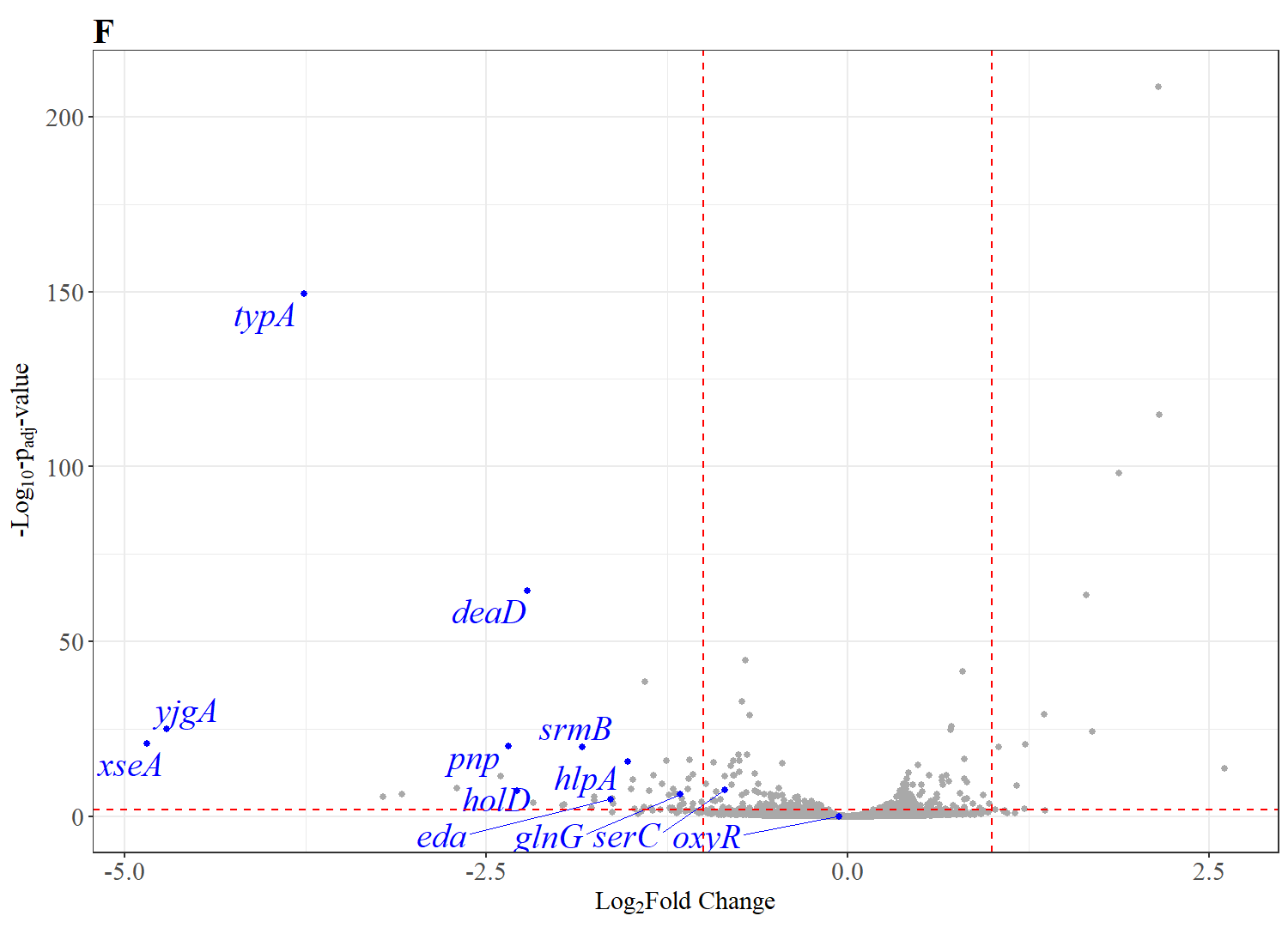


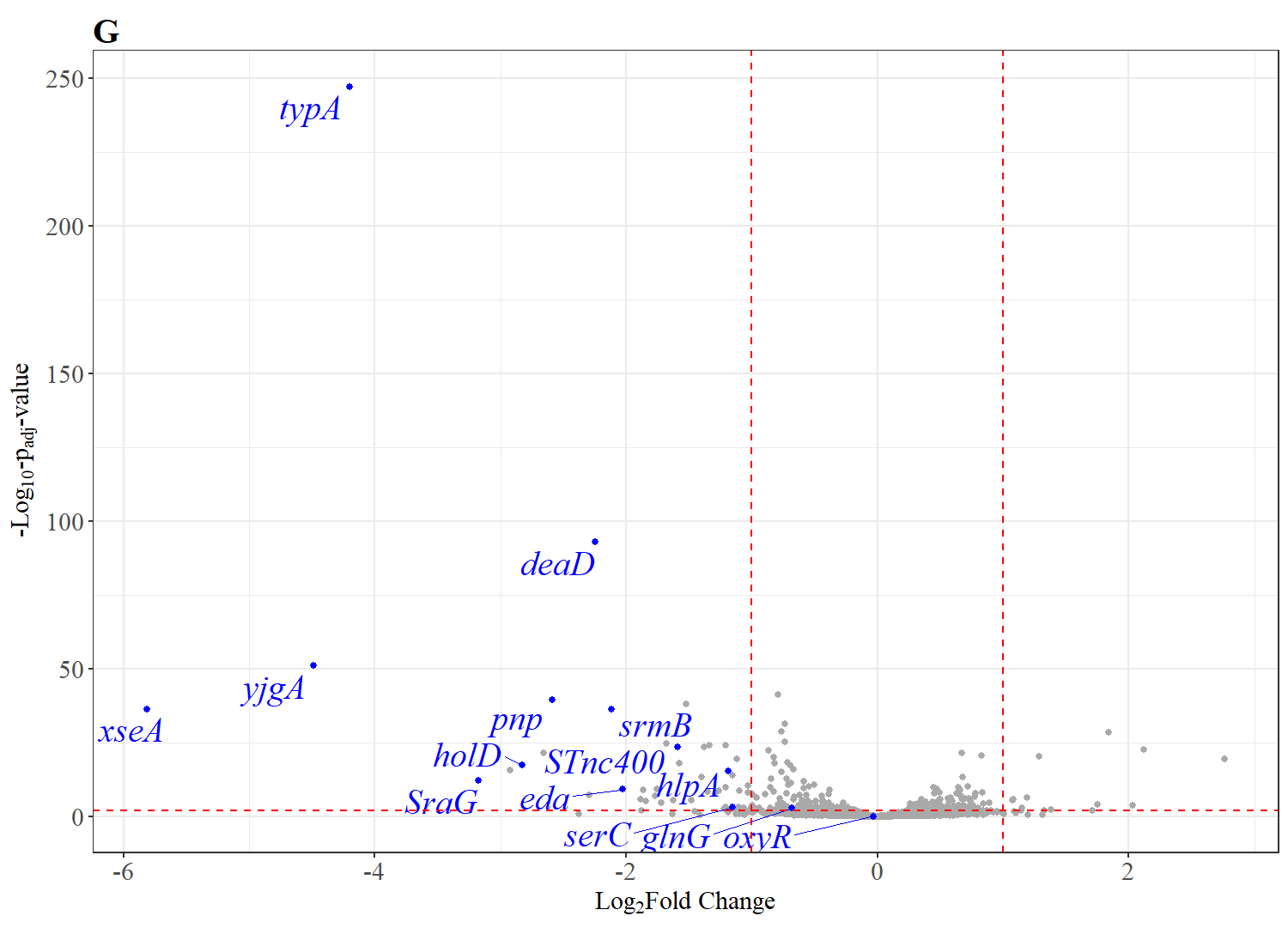


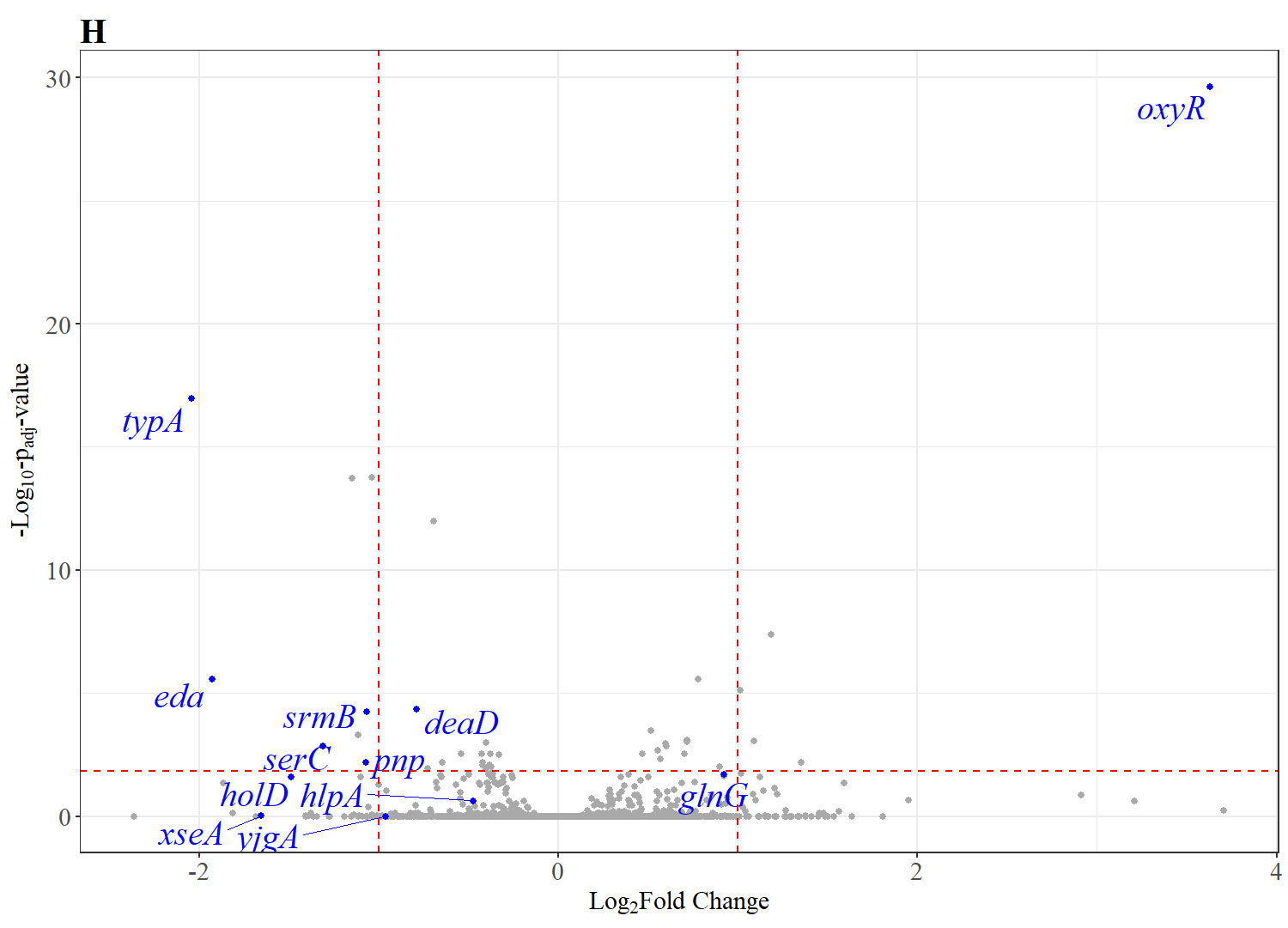


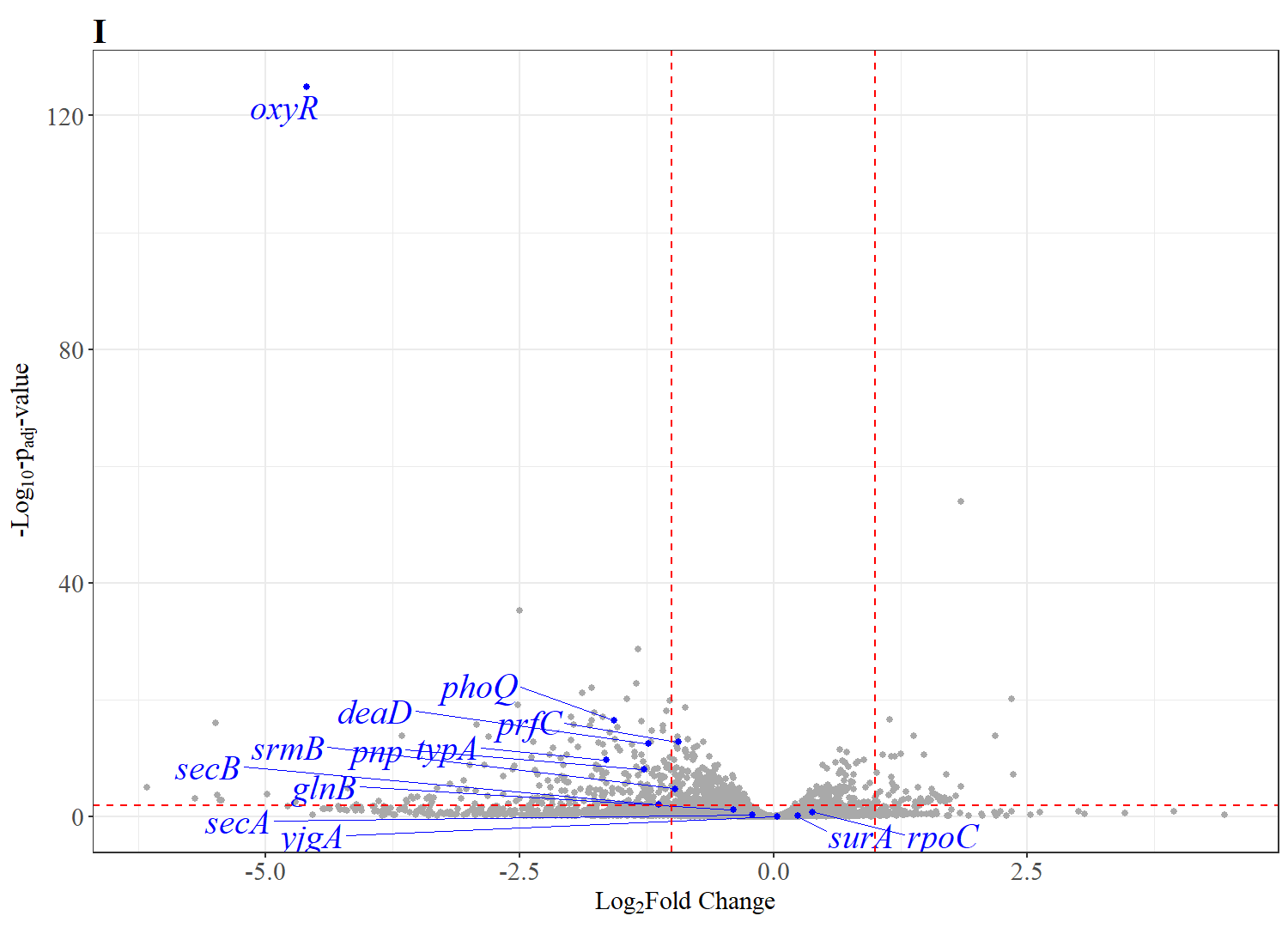


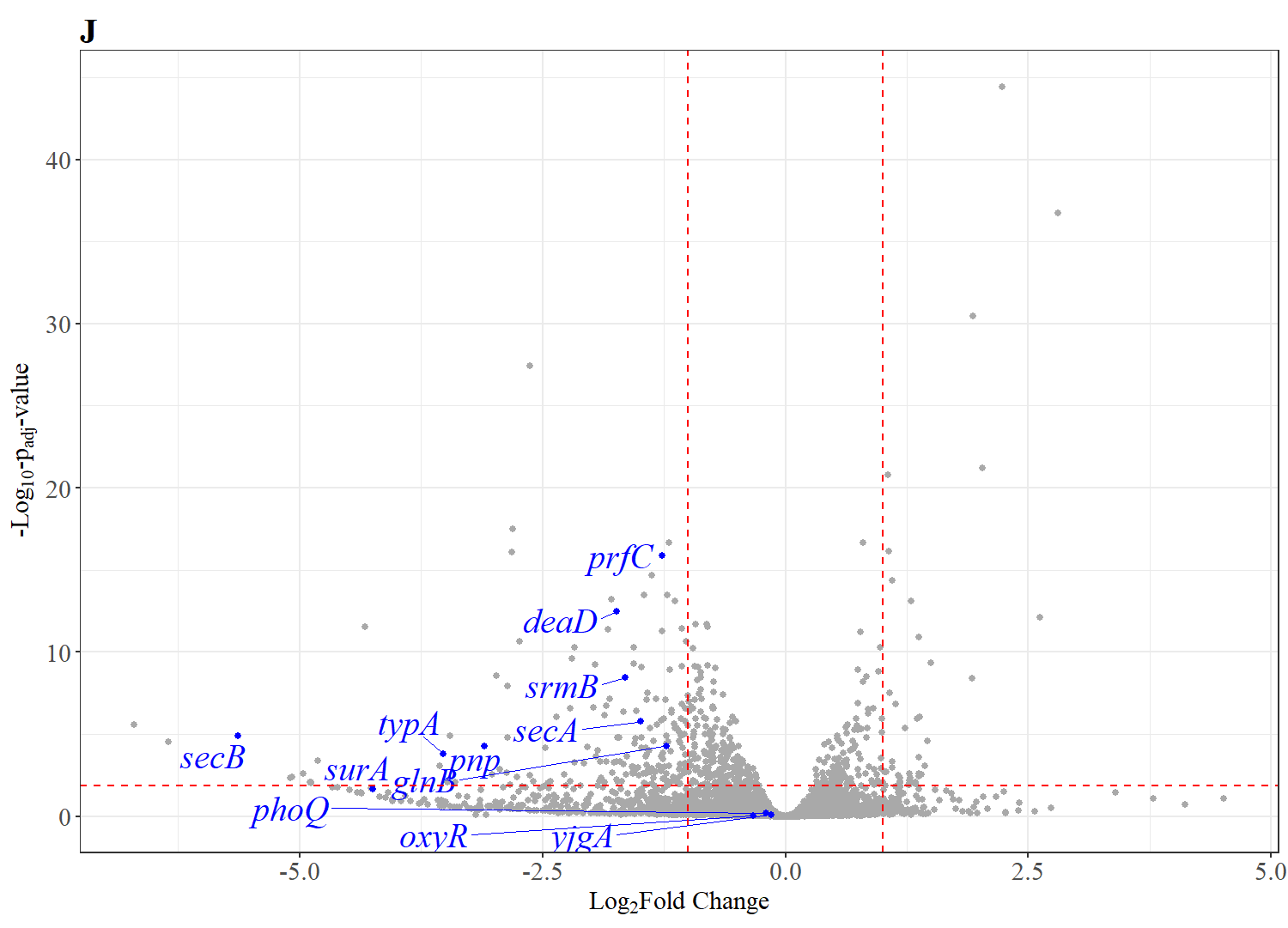


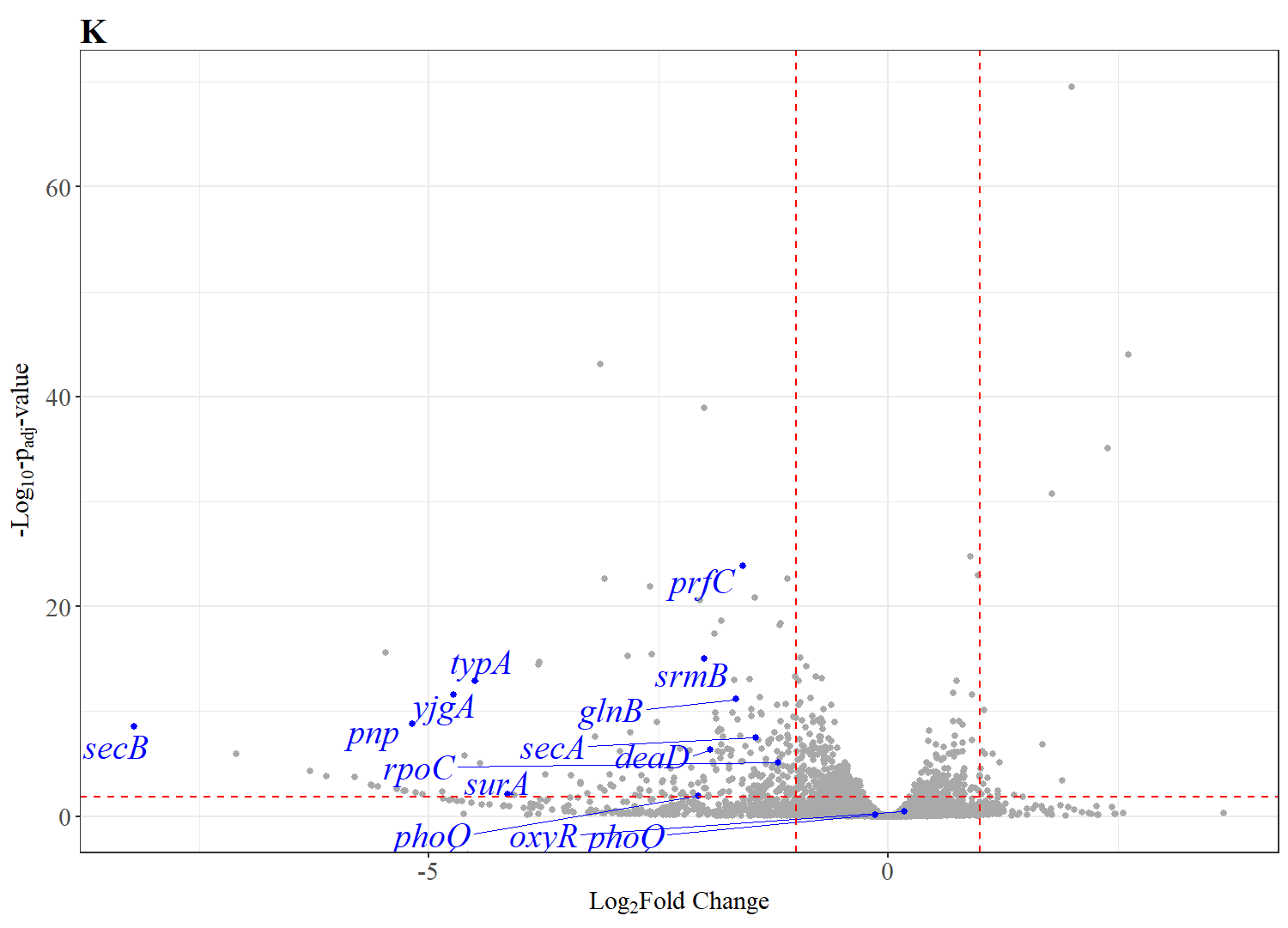


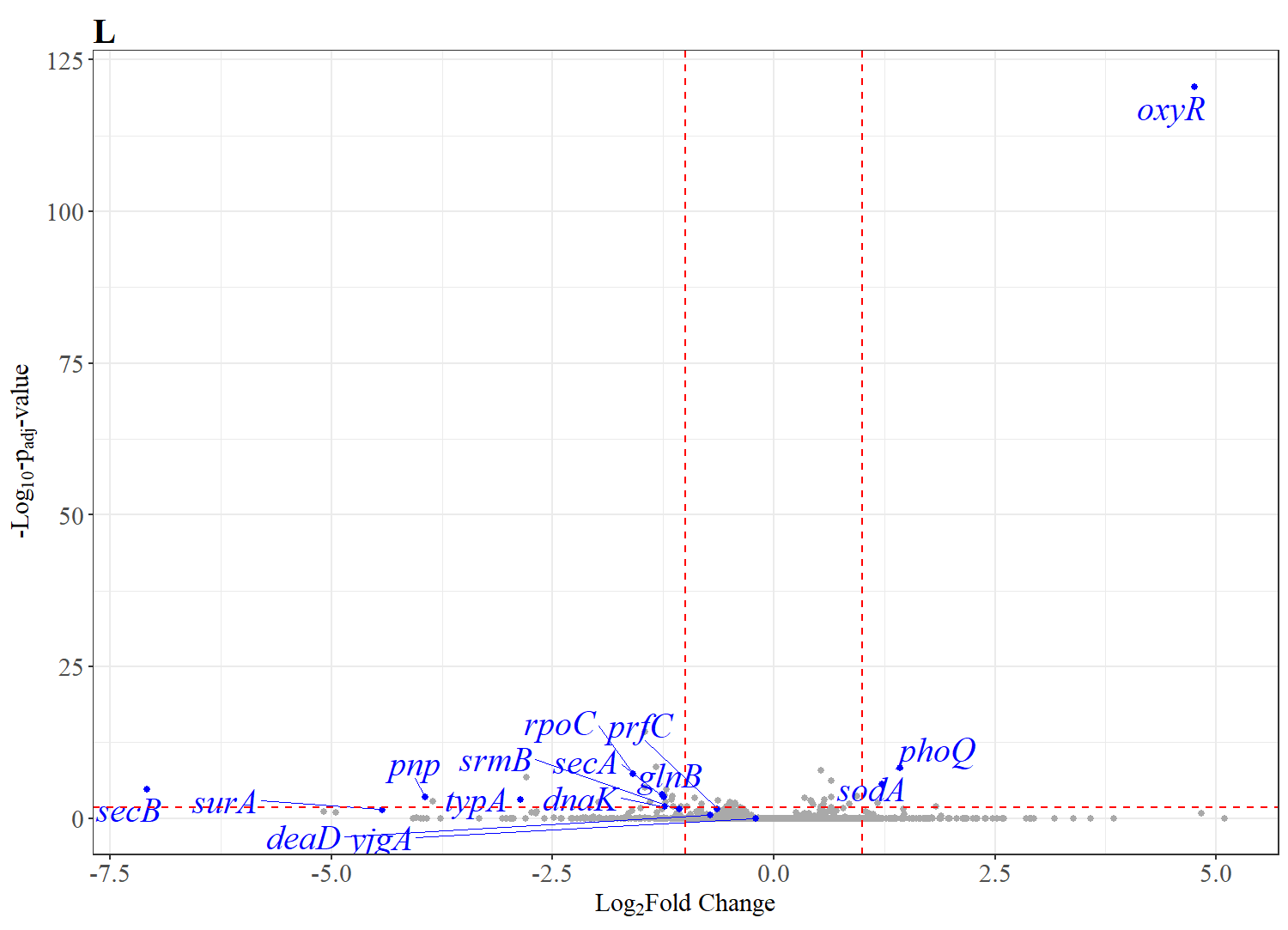


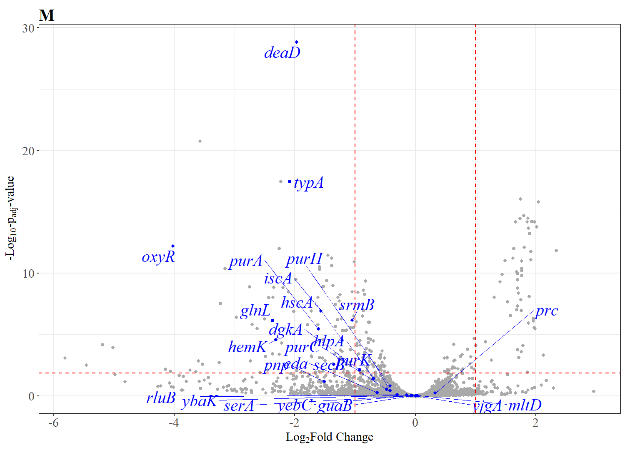


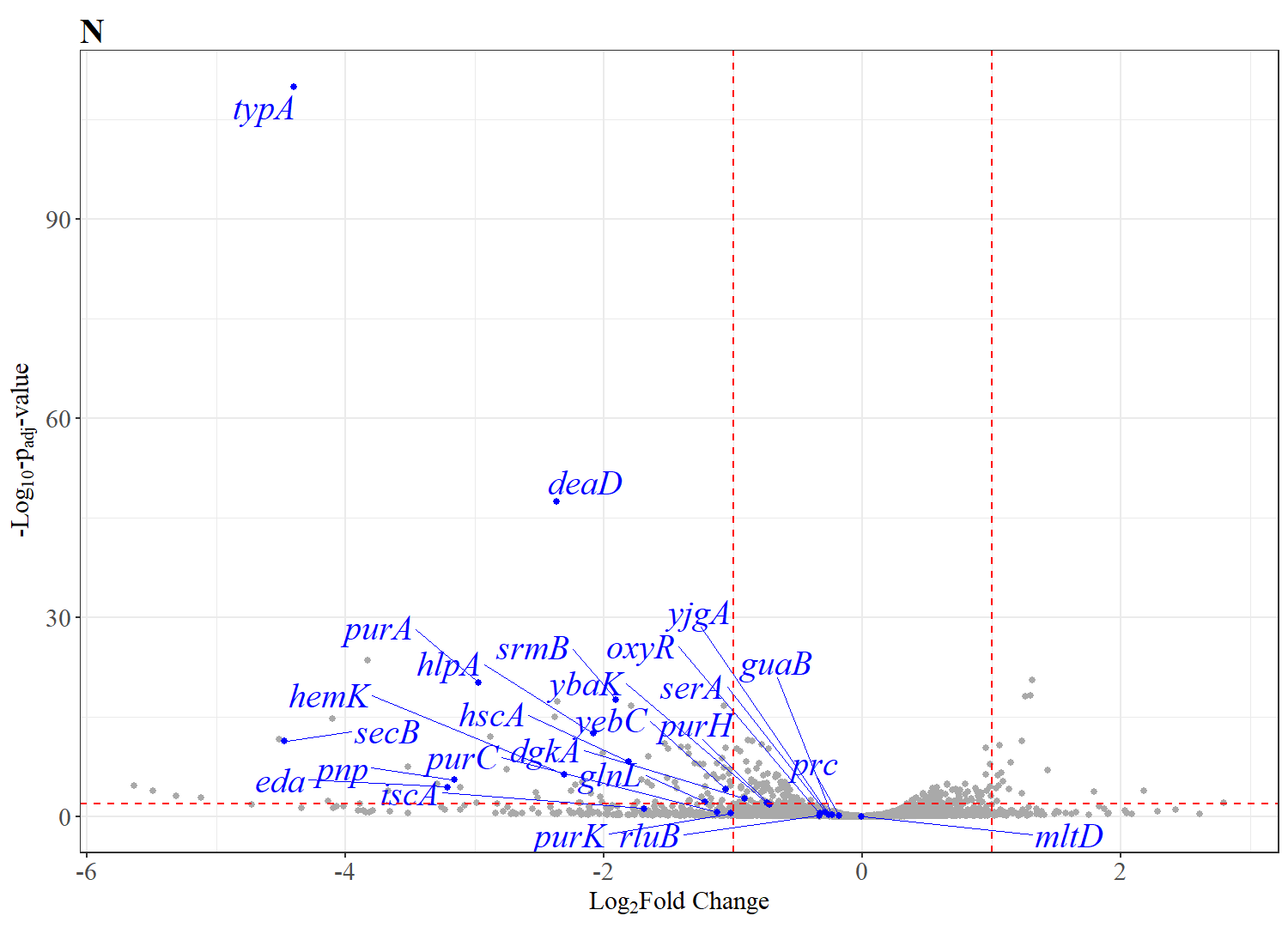


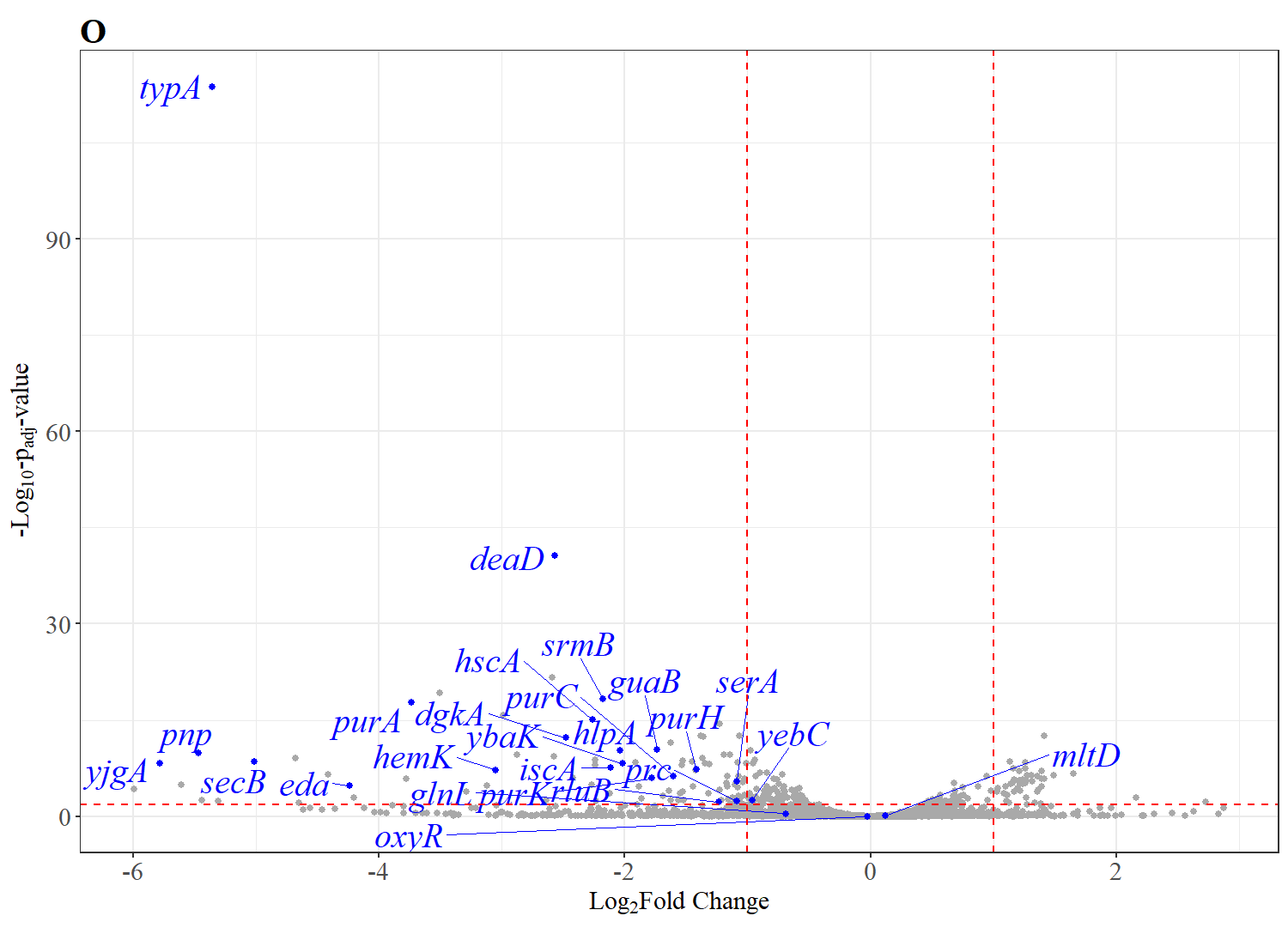


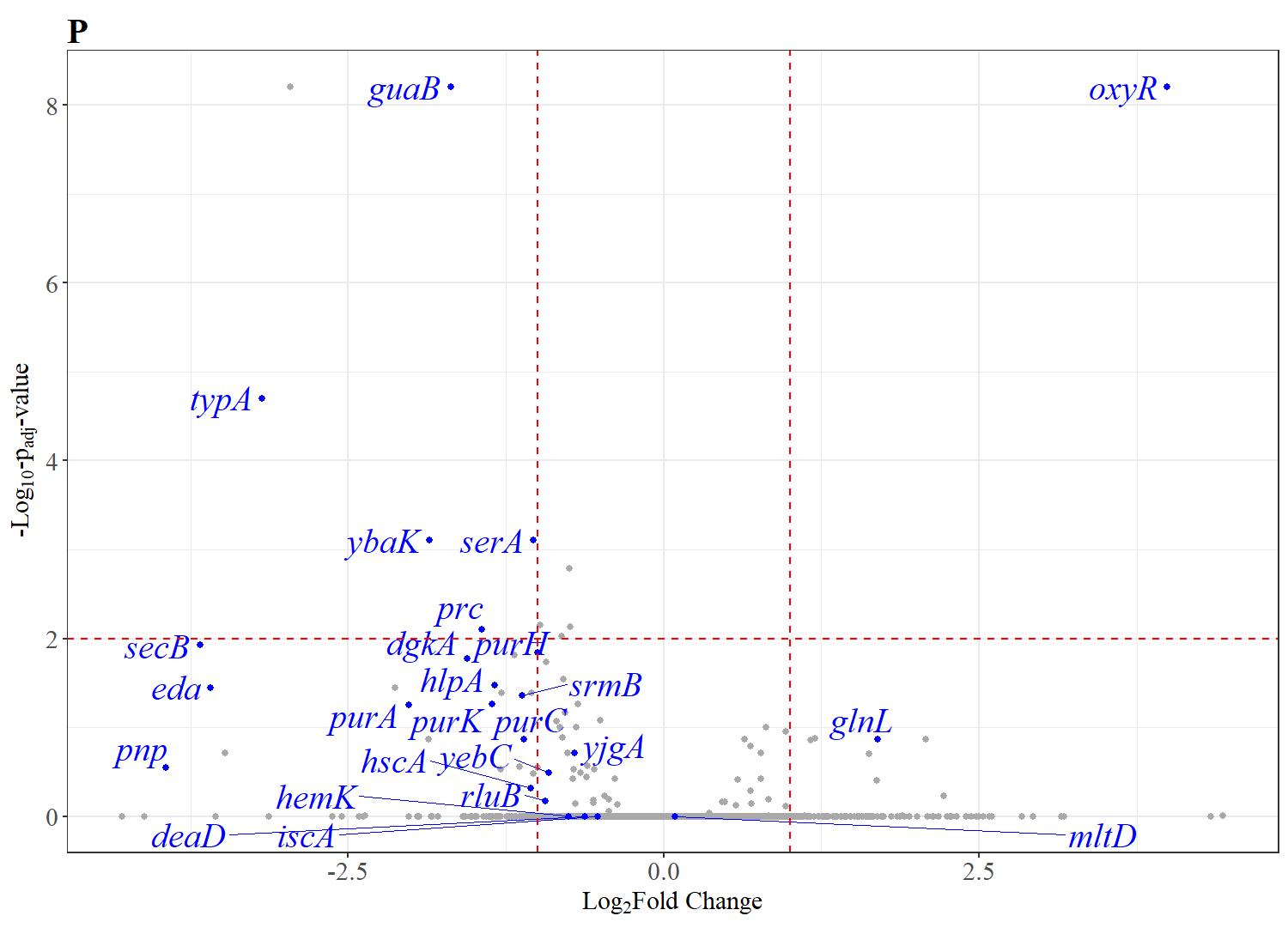


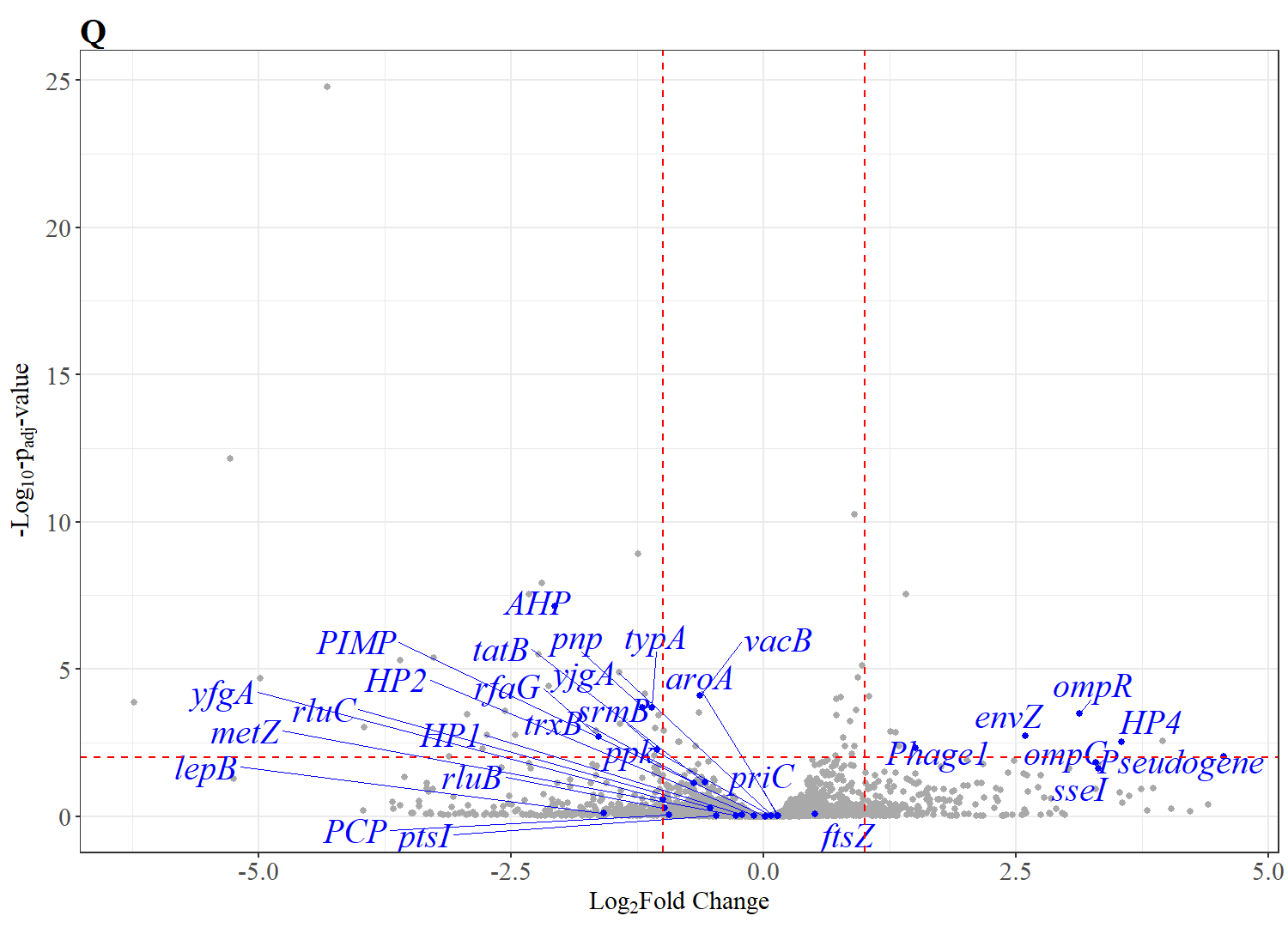


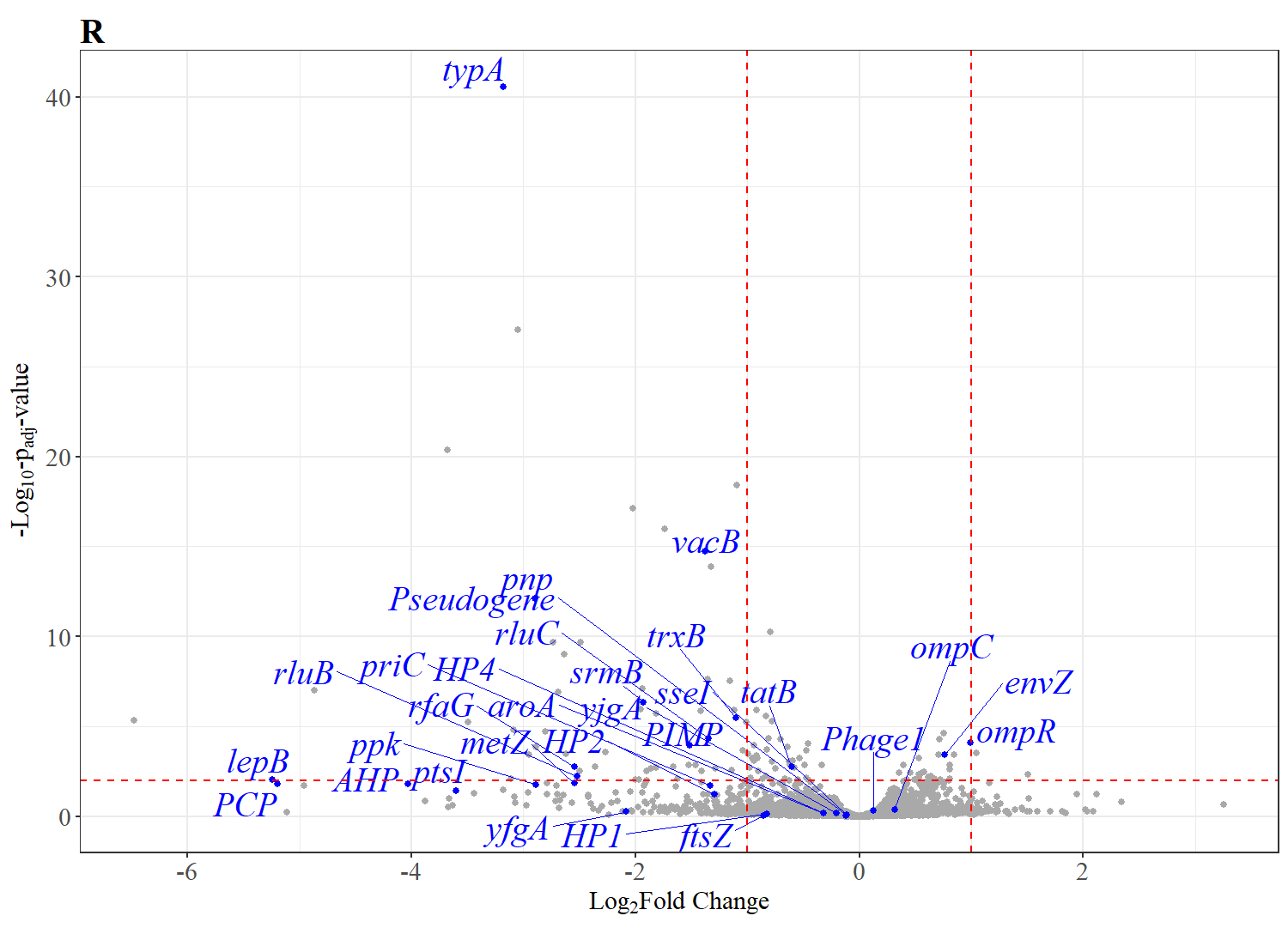


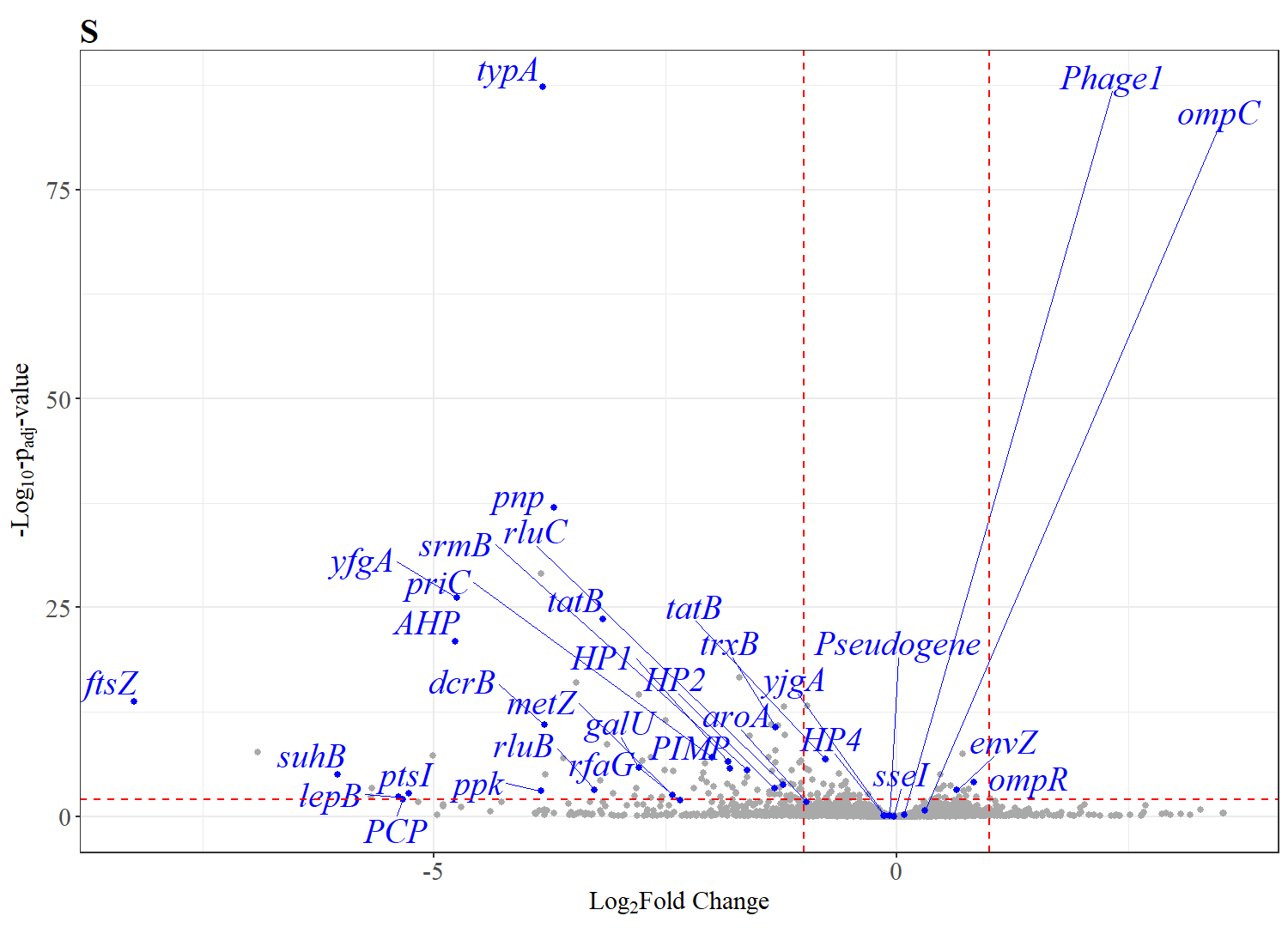


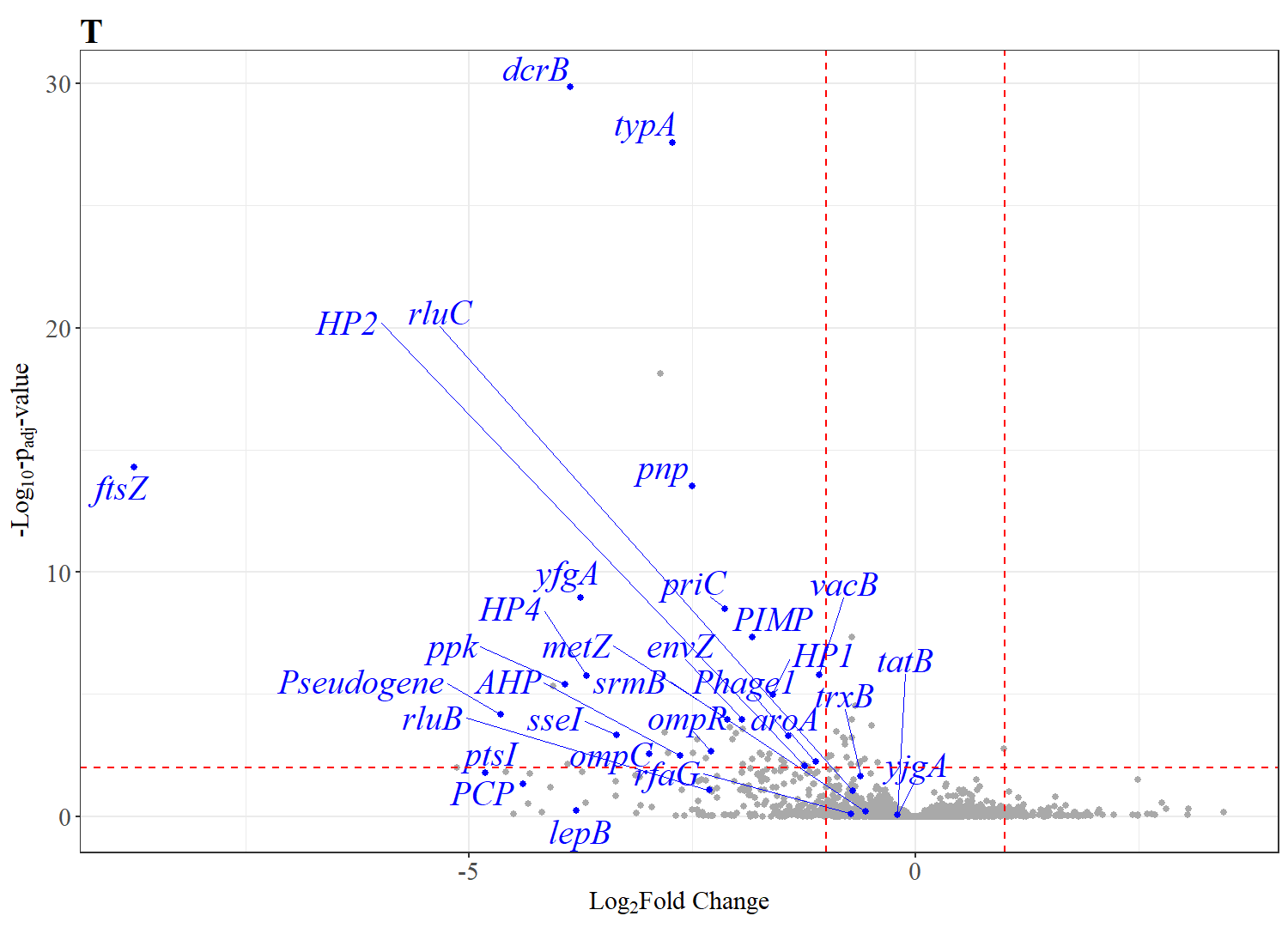


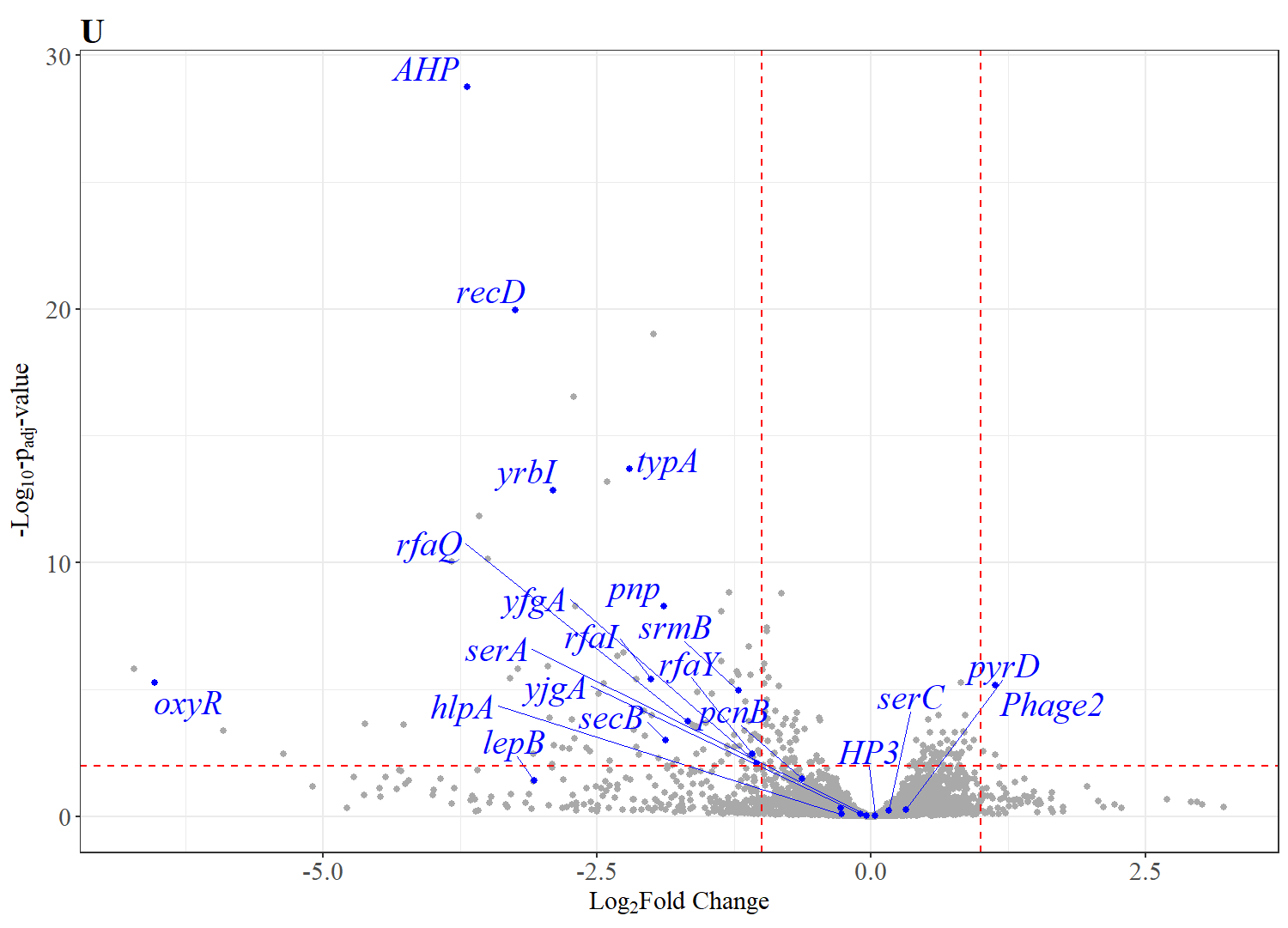


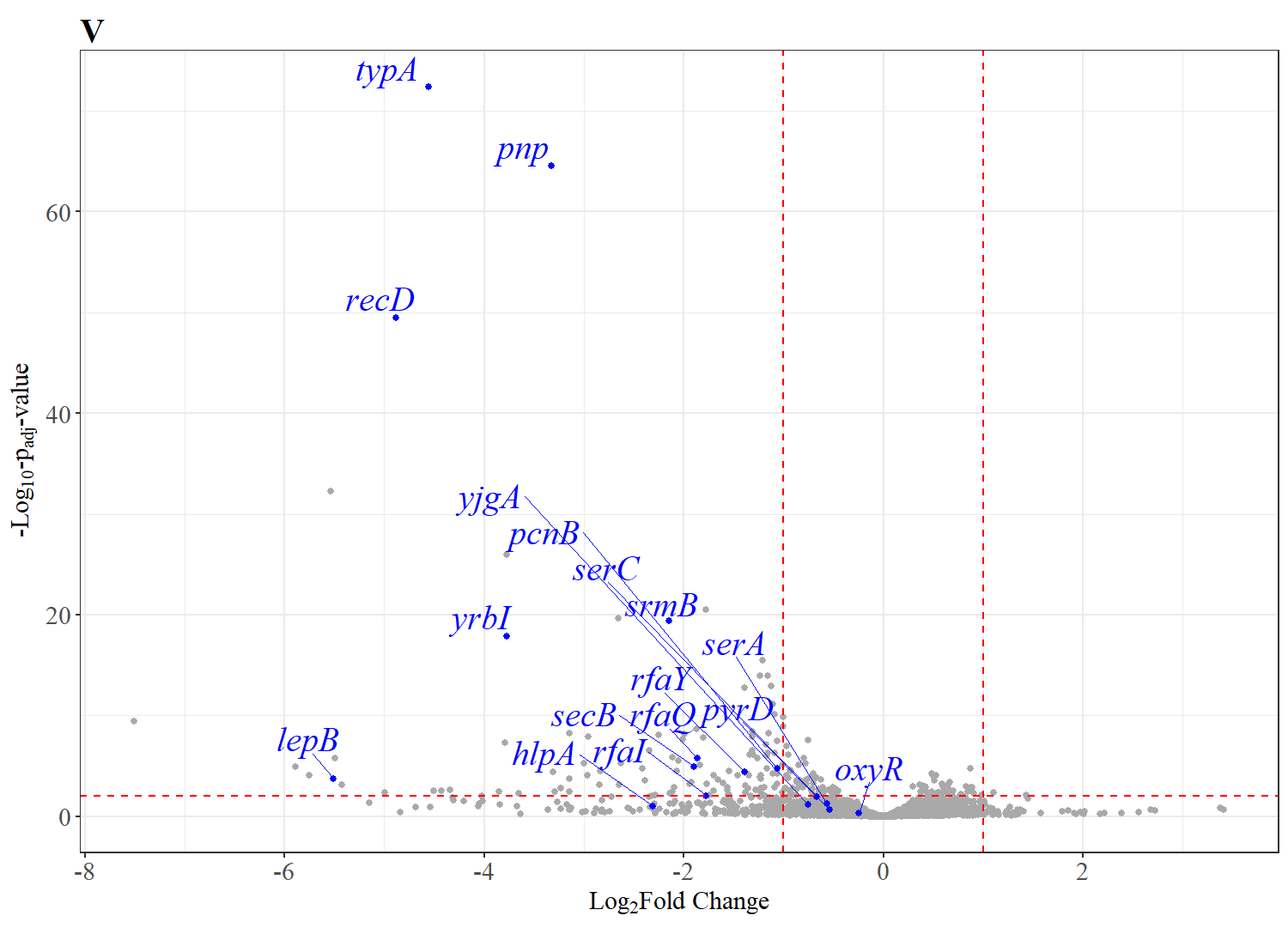


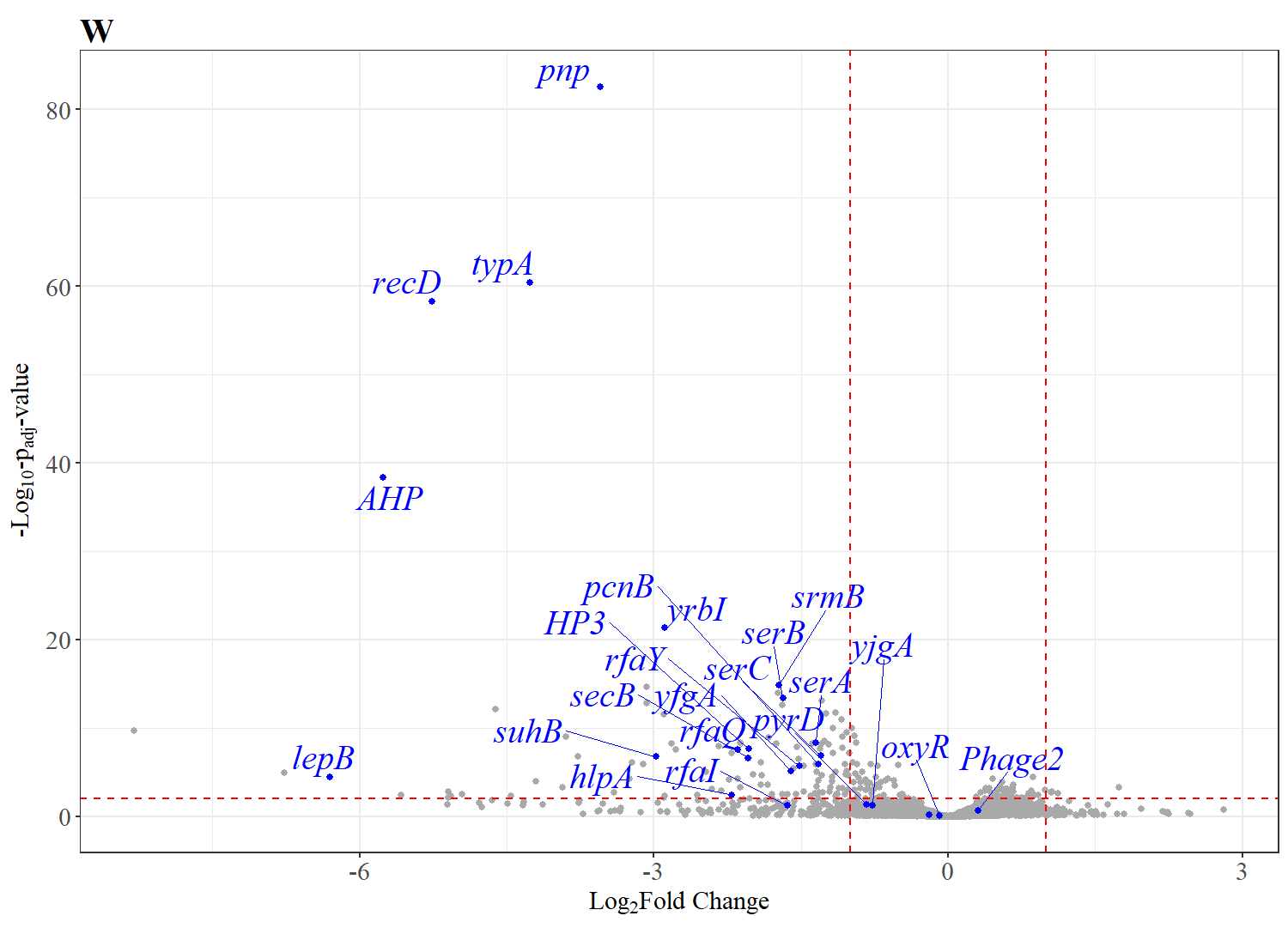


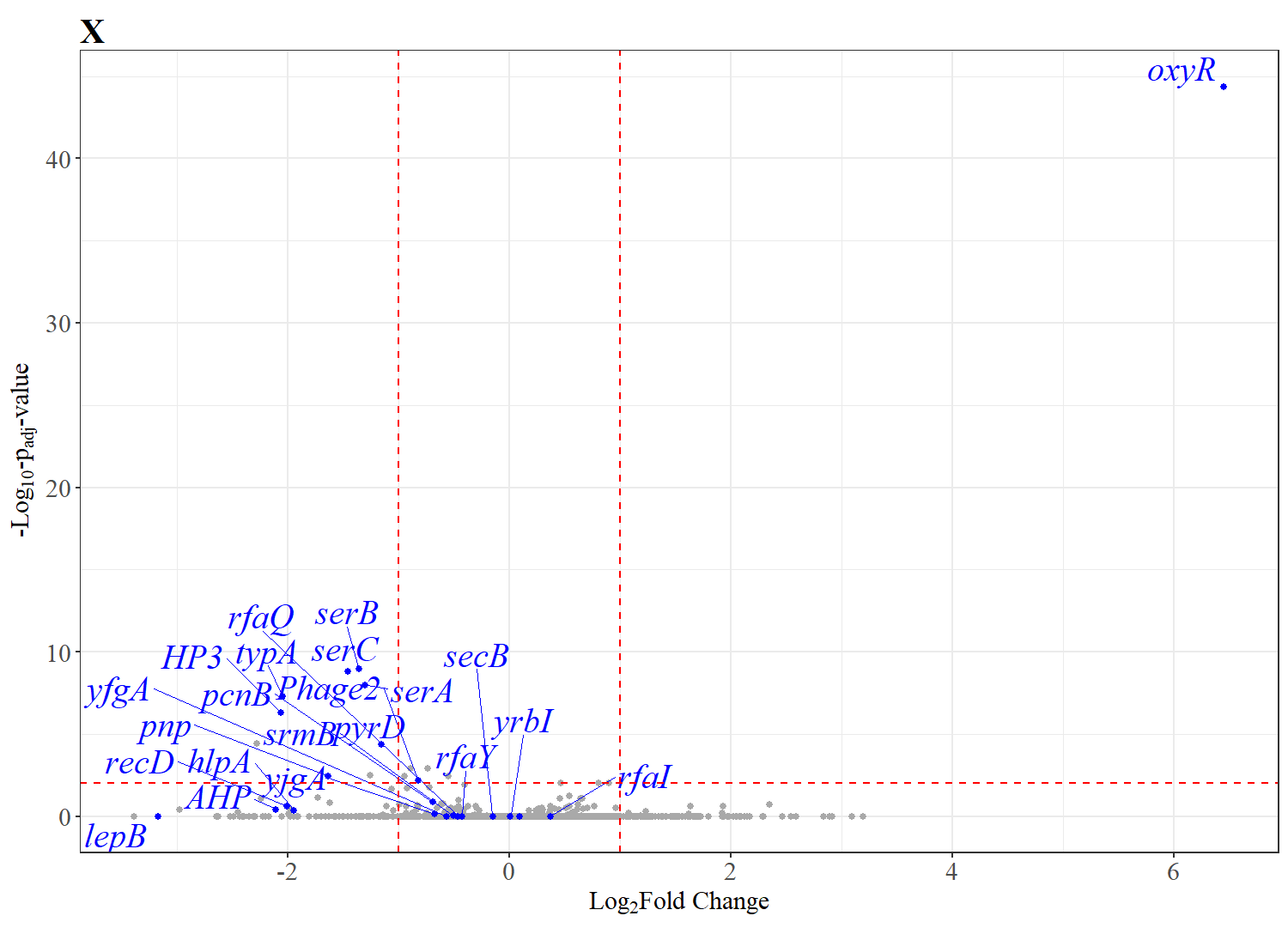


**Supplementary Figure S4.** **Changes in the aggregated mutant abundances throughout the interaction period of the barcoded transposon mutant libraries on ready-to-eat muskmelon.** Volcano plots depict the fold change of relative abundance of mutants and their corresponding adjusted *p*-values between sampling time points for *S.* Typhimurium 14028s **(A-H)**, *S.* Enteritidis PT4 strain P125109 **(I-P)**, and *S.* Newport C4.2 **(Q-X)**. The represented interaction periods at 8°C include inoculum vs 1 h post- inoculation **(I-d_1_; A, I, and Q)**; inoculum vs 48 h **(I-d_3_; B, J, R)**; inoculum vs 96 h **(I-d5, C, K, S)**; and **d_1_-d_5_** **(D, L, T)**. For 22°C, the following interaction periods were included: inoculum vs 1 h post-inoculation **(I-t_1_; E, M, U)**, inoculum vs 7 h **(I-t_7_; F, N, V)**, inoculum and 24 h **(I-t_24_; G, O, W)**; and **t_1_-t_24_ (H, P, X)**. Genes with significant fitness effects included in Table 2 are labelled in blue. Intergenic regions with significant fitness effects were excluded from the plots.
