# Supplementary material for: Genomic factors contributing to the resilience of Salmonella enterica on ready-to-eat muskmelon
Source: Food Microbiol. Author manuscript; Available in PMC 2026 Mar 1. (PMC12767474; doi:10.1016/j.fm.2025.104947)
Supplement: MMC 5 [file NIHMS2127750-supplement-MMC_5.docx]

**Supplementary Table S1. Primers employed for library preparation prior to Illumina sequencing.**

| L501_TCGCCTTA | AATGATACGGCGACCACCGAGATCTACACTCGCCTTAACACTCTTTCCCTACACGACGCTCTTCCGATCTACTCACTATAGGGAGACCGGCCT |
| --- | --- |
| L502_CTAGTACG | AATGATACGGCGACCACCGAGATCTACACCTAGTACGACACTCTTTCCCTACACGACGCTCTTCCGATCTACTCACTATAGGGAGACCGGCCT |
| L503_TTCTGCCT | AATGATACGGCGACCACCGAGATCTACACTTCTGCCTACACTCTTTCCCTACACGACGCTCTTCCGATCTACTCACTATAGGGAGACCGGCCT |
| L504_GCTCAGGA | AATGATACGGCGACCACCGAGATCTACACGCTCAGGAACACTCTTTCCCTACACGACGCTCTTCCGATCTACTCACTATAGGGAGACCGGCCT |
| L505_AGGAGTCC | AATGATACGGCGACCACCGAGATCTACACAGGAGTCCACACTCTTTCCCTACACGACGCTCTTCCGATCTACTCACTATAGGGAGACCGGCCT |
| L506_CATGCCTA | AATGATACGGCGACCACCGAGATCTACACCATGCCTAACACTCTTTCCCTACACGACGCTCTTCCGATCTACTCACTATAGGGAGACCGGCCT |
| L507_GTAGAGAG | AATGATACGGCGACCACCGAGATCTACACGTAGAGAGACACTCTTTCCCTACACGACGCTCTTCCGATCTACTCACTATAGGGAGACCGGCCT |
| L508_CCTCTCTG | AATGATACGGCGACCACCGAGATCTACACCCTCTCTGACACTCTTTCCCTACACGACGCTCTTCCGATCTACTCACTATAGGGAGACCGGCCT |
| L509_AGCGTAGC | AATGATACGGCGACCACCGAGATCTACACAGCGTAGCACACTCTTTCCCTACACGACGCTCTTCCGATCTACTCACTATAGGGAGACCGGCCT |
| L510_CAGCCTCG | AATGATACGGCGACCACCGAGATCTACACCAGCCTCGACACTCTTTCCCTACACGACGCTCTTCCGATCTACTCACTATAGGGAGACCGGCCT |
| L511_TGCCTCTT | AATGATACGGCGACCACCGAGATCTACACTGCCTCTTACACTCTTTCCCTACACGACGCTCTTCCGATCTACTCACTATAGGGAGACCGGCCT |
| L512_TCCTCTAC | AATGATACGGCGACCACCGAGATCTACACTCCTCTACACACTCTTTCCCTACACGACGCTCTTCCGATCTACTCACTATAGGGAGACCGGCCT |
| L513_TCATGAGC | AATGATACGGCGACCACCGAGATCTACACTCATGAGCACACTCTTTCCCTACACGACGCTCTTCCGATCTACTCACTATAGGGAGACCGGCCT |
| L514_CCTGAGAT | AATGATACGGCGACCACCGAGATCTACACCCTGAGATACACTCTTTCCCTACACGACGCTCTTCCGATCTACTCACTATAGGGAGACCGGCCT |
| L515_TAGCGAGT | AATGATACGGCGACCACCGAGATCTACACTAGCGAGTACACTCTTTCCCTACACGACGCTCTTCCGATCTACTCACTATAGGGAGACCGGCCT |
| L516_GTAGCTCC | AATGATACGGCGACCACCGAGATCTACACGTAGCTCCACACTCTTTCCCTACACGACGCTCTTCCGATCTACTCACTATAGGGAGACCGGCCT |
| L517_TACTACGC | AATGATACGGCGACCACCGAGATCTACACTACTACGCACACTCTTTCCCTACACGACGCTCTTCCGATCTACTCACTATAGGGAGACCGGCCT |
| L518_GCAGCGTA | AATGATACGGCGACCACCGAGATCTACACGCAGCGTAACACTCTTTCCCTACACGACGCTCTTCCGATCTACTCACTATAGGGAGACCGGCCT |
| L519_CTGCGCAT | AATGATACGGCGACCACCGAGATCTACACCTGCGCATACACTCTTTCCCTACACGACGCTCTTCCGATCTACTCACTATAGGGAGACCGGCCT |
| L520_GAGCGCTA | AATGATACGGCGACCACCGAGATCTACACGAGCGCTAACACTCTTTCCCTACACGACGCTCTTCCGATCTACTCACTATAGGGAGACCGGCCT |
| L521_CGCTCAGT | AATGATACGGCGACCACCGAGATCTACACCGCTCAGTACACTCTTTCCCTACACGACGCTCTTCCGATCTACTCACTATAGGGAGACCGGCCT |
| L522_GTCTTAGG | AATGATACGGCGACCACCGAGATCTACACGTCTTAGGACACTCTTTCCCTACACGACGCTCTTCCGATCTACTCACTATAGGGAGACCGGCCT |
| L523_ACTGATCG | AATGATACGGCGACCACCGAGATCTACACACTGATCGACACTCTTTCCCTACACGACGCTCTTCCGATCTACTCACTATAGGGAGACCGGCCT |
| L524_TAGCTGCA | AATGATACGGCGACCACCGAGATCTACACTAGCTGCAACACTCTTTCCCTACACGACGCTCTTCCGATCTACTCACTATAGGGAGACCGGCCT |
| V701_CTCTCTAT | CAAGCAGAAGACGGCATACGAGATCTCTCTATGTGACTGGAGTTCAGACGTGTGCTCTTCCGATCTGTCTCTTATACACATCTCAACCCTG |
| V702_TATCCTCT | CAAGCAGAAGACGGCATACGAGATTATCCTCTGTGACTGGAGTTCAGACGTGTGCTCTTCCGATCTGTCTCTTATACACATCTCAACCCTG |
| V703_GTAAGGAG | CAAGCAGAAGACGGCATACGAGATGTAAGGAGGTGACTGGAGTTCAGACGTGTGCTCTTCCGATCTGTCTCTTATACACATCTCAACCCTG |
| V704_ACTGCATA | CAAGCAGAAGACGGCATACGAGATACTGCATAGTGACTGGAGTTCAGACGTGTGCTCTTCCGATCTGTCTCTTATACACATCTCAACCCTG |
| V705_AAGGAGTA | CAAGCAGAAGACGGCATACGAGATAAGGAGTAGTGACTGGAGTTCAGACGTGTGCTCTTCCGATCTGTCTCTTATACACATCTCAACCCTG |
| V706_CTAAGCCT | CAAGCAGAAGACGGCATACGAGATCTAAGCCTGTGACTGGAGTTCAGACGTGTGCTCTTCCGATCTGTCTCTTATACACATCTCAACCCTG |
| V707_CGTCTAAT | CAAGCAGAAGACGGCATACGAGATCGTCTAATGTGACTGGAGTTCAGACGTGTGCTCTTCCGATCTGTCTCTTATACACATCTCAACCCTG |
| V708_TCTCTCCG | CAAGCAGAAGACGGCATACGAGATTCTCTCCGGTGACTGGAGTTCAGACGTGTGCTCTTCCGATCTGTCTCTTATACACATCTCAACCCTG |
| V709_TCGACTAG | CAAGCAGAAGACGGCATACGAGATTCGACTAGGTGACTGGAGTTCAGACGTGTGCTCTTCCGATCTGTCTCTTATACACATCTCAACCCTG |
| V710_TTCTAGCT | CAAGCAGAAGACGGCATACGAGATTTCTAGCTGTGACTGGAGTTCAGACGTGTGCTCTTCCGATCTGTCTCTTATACACATCTCAACCCTG |
| V711_CCTAGAGT | CAAGCAGAAGACGGCATACGAGATCCTAGAGTGTGACTGGAGTTCAGACGTGTGCTCTTCCGATCTGTCTCTTATACACATCTCAACCCTG |
| V712_GCGTAAGA | CAAGCAGAAGACGGCATACGAGATGCGTAAGAGTGACTGGAGTTCAGACGTGTGCTCTTCCGATCTGTCTCTTATACACATCTCAACCCTG |
| V713_CTATTAAG | CAAGCAGAAGACGGCATACGAGATCTATTAAGGTGACTGGAGTTCAGACGTGTGCTCTTCCGATCTGTCTCTTATACACATCTCAACCCTG |
| V714_AAGGCTAT | CAAGCAGAAGACGGCATACGAGATAAGGCTATGTGACTGGAGTTCAGACGTGTGCTCTTCCGATCTGTCTCTTATACACATCTCAACCCTG |
| V715_GAGCCTTA | CAAGCAGAAGACGGCATACGAGATGAGCCTTAGTGACTGGAGTTCAGACGTGTGCTCTTCCGATCTGTCTCTTATACACATCTCAACCCTG |
| V716_TTATGCGA | CAAGCAGAAGACGGCATACGAGATTTATGCGAGTGACTGGAGTTCAGACGTGTGCTCTTCCGATCTGTCTCTTATACACATCTCAACCCTG |
| V717_AGCGTGCG | CAAGCAGAAGACGGCATACGAGATAGCGTGCGGTGACTGGAGTTCAGACGTGTGCTCTTCCGATCTGTCTCTTATACACATCTCAACCCTG |
| V718_AACCGCTC | CAAGCAGAAGACGGCATACGAGATAACCGCTCGTGACTGGAGTTCAGACGTGTGCTCTTCCGATCTGTCTCTTATACACATCTCAACCCTG |
| V719_GACAACGC | CAAGCAGAAGACGGCATACGAGATGACAACGCGTGACTGGAGTTCAGACGTGTGCTCTTCCGATCTGTCTCTTATACACATCTCAACCCTG |
| V720_AGCCAAGC | CAAGCAGAAGACGGCATACGAGATAGCCAAGCGTGACTGGAGTTCAGACGTGTGCTCTTCCGATCTGTCTCTTATACACATCTCAACCCTG |
| V721_GGATGTCA | CAAGCAGAAGACGGCATACGAGATGGATGTCAGTGACTGGAGTTCAGACGTGTGCTCTTCCGATCTGTCTCTTATACACATCTCAACCCTG |
| V722_TCGGTGGC | CAAGCAGAAGACGGCATACGAGATTCGGTGGCGTGACTGGAGTTCAGACGTGTGCTCTTCCGATCTGTCTCTTATACACATCTCAACCCTG |
| V723_GGACTCTC | CAAGCAGAAGACGGCATACGAGATGGACTCTCGTGACTGGAGTTCAGACGTGTGCTCTTCCGATCTGTCTCTTATACACATCTCAACCCTG |
| V724_CGTAAGTG | CAAGCAGAAGACGGCATACGAGATCGTAAGTGGTGACTGGAGTTCAGACGTGTGCTCTTCCGATCTGTCTCTTATACACATCTCAACCCTG |
